# FAIR data station for lightweight metadata management and validation of omics studies

**DOI:** 10.1093/gigascience/giad014

**Published:** 2023-03-06

**Authors:** Bart Nijsse, Peter J Schaap, Jasper J Koehorst

**Affiliations:** Laboratory of Systems and Synthetic Biology, Wageningen University & Research, Stippeneng 4, 6708 WE Wageningen, The Netherlands; UNLOCK Large Scale Infrastructure for Microbial Communities, Wageningen University & Research and Delft University of Technology, Stippeneng 4, 6708 WE Wageningen, The Netherlands; Laboratory of Systems and Synthetic Biology, Wageningen University & Research, Stippeneng 4, 6708 WE Wageningen, The Netherlands; UNLOCK Large Scale Infrastructure for Microbial Communities, Wageningen University & Research and Delft University of Technology, Stippeneng 4, 6708 WE Wageningen, The Netherlands; Laboratory of Systems and Synthetic Biology, Wageningen University & Research, Stippeneng 4, 6708 WE Wageningen, The Netherlands; UNLOCK Large Scale Infrastructure for Microbial Communities, Wageningen University & Research and Delft University of Technology, Stippeneng 4, 6708 WE Wageningen, The Netherlands

**Keywords:** FAIR, metadata, MIxS standards, ENA submission tool, semantic web, ontologies

## Abstract

**Background:**

The life sciences are one of the biggest suppliers of scientific data. Reusing and connecting these data can uncover hidden insights and lead to new concepts. Efficient reuse of these datasets is strongly promoted when they are interlinked with a sufficient amount of machine-actionable metadata. While the FAIR (Findable, Accessible, Interoperable, Reusable) guiding principles have been accepted by all stakeholders, in practice, there are only a limited number of easy-to-adopt implementations available that fulfill the needs of data producers.

**Findings:**

We developed the FAIR Data Station, a lightweight application written in Java, that aims to support researchers in managing research metadata according to the FAIR principles. It implements the ISA metadata framework and uses minimal information metadata standards to capture experiment metadata. The FAIR Data Station consists of 3 modules. Based on the minimal information model(s) selected by the user, the “form generation module” creates a metadata template Excel workbook with a header row of machine-actionable attribute names. The Excel workbook is subsequently used by the data producer(s) as a familiar environment for sample metadata registration. At any point during this process, the format of the recorded values can be checked using the “validation module.” Finally, the “resource module” can be used to convert the set of metadata recorded in the Excel workbook in RDF format, enabling (cross-project) (meta)data searches and, for publishing of sequence data, in an European Nucleotide Archive–compatible XML metadata file.

**Conclusions:**

Turning FAIR into reality requires the availability of easy-to-adopt data FAIRification workflows that are also of direct use for data producers. As such, the FAIR Data Station provides, in addition to the means to correctly FAIRify (omics) data, the means to build searchable metadata databases of similar projects and can assist in ENA metadata submission of sequence data. The FAIR Data Station is available at https://fairbydesign.nl.

## Background

Online repositories sharing scientific data are vital for the advancement of science. Data sharing improves research transparency, promotes the validation of experimental methods and scientific conclusions, enables data reuse, and facilitates knowledge discovery using new analysis tools. Essential for reusing scientific data is the availability of machine-readable metadata about the scientific experiments conducted with a degree of completeness that reflects the FAIR guiding principles: Findable, Accessible, Interoperable, Reusable [1].

Several tools have been created to help make data FAIR. The ISA metadata framework standard [2] outlines a model for capturing experiment metadata using 3 levels: Investigation, Study, and Assay. The FAIRDOM Hub uses the ISA framework to create a collaboration platform for systems biology research, but it does not offer high-throughput validation [3]. The GO-FAIR initiative outlines a 7-step workflow for making data FAIR but does not include practical implementations for the technology needed [4]. Note that FAIR is not a standard but a set of guidelines that can be interpreted differently.

A key feature of properly FAIRified data is a high level of data interoperability. From a data producer/user point of view, 2 levels are important: structural and semantic interoperability. Structural interoperability defines the format of the data, allowing the data to be interpreted by multiple systems. For example, the FASTA sequence format is the most implemented and best machine-actionable data standard for sequence data and therefore directly understood by many sequence analysis tools [5, 6]. Semantic interoperability entails the transformation of ambiguous human-understandable metadata in a standardized machine-actionable open format, allowing computational support systems to automatically find, access, and reuse data. To ensure that the set of metadata is sufficient for the data to be unambiguously described, standardized minimal information models and checklists, detailing those requirements, have been developed for wide array of experiment data [7].

Next-generation high-throughput sequencing experiments are the major big data generators of the life sciences [8]. Sequence data are a special case as they imply a large-scale assessment of a single type of molecules. This property and its representation in standard FASTA format make the sequence data type an excellent candidate for data reuse. To assist in the FAIRification process of sequence data, the Genomic Standards Consortium [9] has developed a widely accepted family of minimum information standard checklists about any (x) Sequence (MIxS). While these guidelines were developed with sequence data in mind, they can also be used to describe sample metadata of other studies.

To help researchers to FAIRify their experiment data in line with accepted standards, we have developed the FAIR Data Station (FAIR-DS). The overall goal of this lightweight stand-alone tool is to assist the domain researcher/data producer in creating high-quality FAIR metadata. The FAIR-DS supports the MIxS set of metadata standards implemented by the main sequence databases such as the European Nucleotide Archive (ENA), Genbank, MGnify (EBI Metagenomics), JGI-GOLD, and others (see https://doi.org/10.25504/FAIRsharing.9aa0zp for more) and can be used to streamline metadata submission of sequence data to ENA. The output of the FAIR-DS can also be directly used to build a metadata database of (similar) projects, while the default set of mandatory and optional metadata fields can easily be expanded to align with the internal standards of a research group.

## Design Considerations

For metadata registration, the FAIR-DS uses an amended version of the original 3-level ISA metadata framework (https://isa-tools.org). The Investigation layer contains human-readable project-related metadata: title, authors, and a minimal amount of high-level information to understand the overall goals of the experiment(s). The Study layer describes a specific research line. As one investigation can have several research lines, each Study layer has a unique user-defined identifier, a study title, and a description of the experimental design of the specific line of research.

As an extension to the original ISA model in between Study and Assay, 2 additional layers of information were added. While developing the tool, we noticed that experimentalists/data producers find the terms “source material” and “sample material” confusingly similar. We therefore implemented the object types “Observation unit,” described in the ISA-Tab format for MIAPPE v1.1 (http://miappe.org) as a replacement for “source material” and implemented the more familiar “Sample” from the Just Enough Results Model (http://jermontology.org). For experimentalists/data producers, adding these 2 layers makes sense as the minimal information models applied focus on contextual data of the sampling environment. The amended schema is aligned with the current ISA model by linking “Observation unit” and “Sample” to the equivalent classes “source material” and “sample material,” respectively.

The number of Observation units used should be in line with the experimental design. The Sample layer describes the conditions under which a biological sample was taken from an Observation unit. A multitude of samples can be taken from a single observational unit. Each of these samples may also be subjected to multiple Assays.

**Figure 1:**
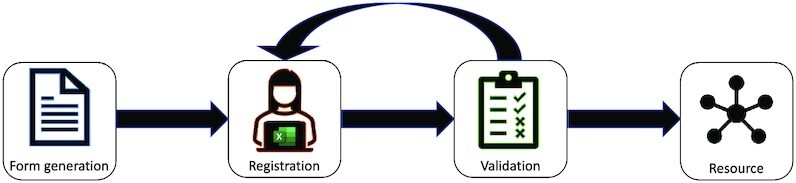
FAIR Data Station metadata registration workflow. The FAIR Data Station workflow consists of 3 main modules. Based on the minimal information checklist(s) selected by the user, the “form generation module” creates a standardized metadata template Excel workbook, and the “validation module” checks the format-restricted metadata recorded in the workbook. The “resource module” exports the complete set of recorded metadata into an RDF data file, enabling (cross-project) metadata searches, and optionally into ENA-compatible metadata submission files.

**Figure 2:**
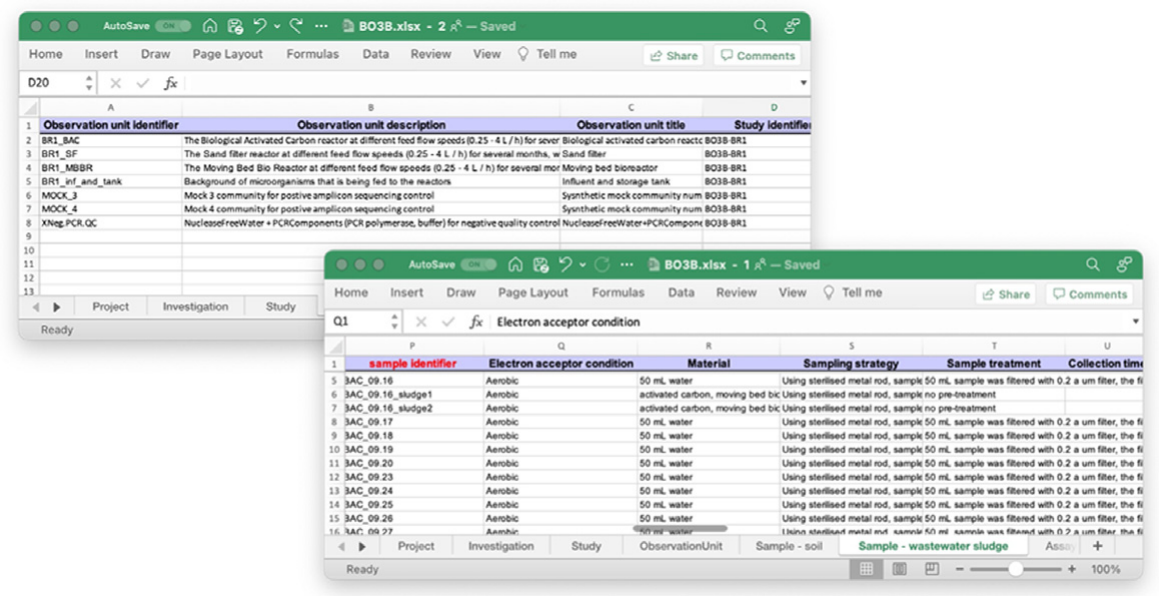
Snapshots of a project metadata workbook generated by the FAIR Data Station showing the Observation unit and Sample worksheets. Column headers represent the mandatory and optional attributes (including instruction notes) selected by the user. Each line represents the metadata values associated with a single observation unit or sample. While the columns are in a default order, they can be rearranged to the user’s preference, and user-defined (comment) columns, such as in this example “Electron acceptor condition,” can be added. User-defined attribute–value pairs are not validated, but user-defined column headers will be used as predicates in the RDF knowledge graph. Note that this is a multisheet workbook in accordance with the ISA standard.

To encourage domain researchers to FAIRify their data in the best possible way, a metadata registration tool should be flexible and require little or no training. For the human-readable high-level metadata registration, we have chosen an intuitive web form. Next, the tool prompts users to choose 1 or more minimal information model(s) that best represent the type of samples taken (Fig. 1). The chosen model(s) specify a set of mandatory and optional attributes that should be used to describe the samples taken. After selection of the most appropriate minimal information model(s) and relevant optional attributes, the FAIR-DS will generate a metadata template workbook in an open Excel format that will allow sample metadata registration in the form of attribute name-value pairs (Fig. 2). The open Excel format was chosen because it allows for offline, on-site metadata registration and supports collaborative efforts and information collection in high throughput. To be able to link different sample types to an observation unit and multiple assay types to a sample, multiple minimal information models can be selected in parallel, which will become available as individual sheets in the workbook.

## Metadata Selection and Validation

To assist domain researchers in creating high-quality FAIR metadata, the FAIR-DS comes with a metadata package library of 40 frequently used minimal information checklists: 23 are MIxS standards [10] not limited to sequence data, and 17 are minimal information checklists directly obtained from ENA [11]. Each individual package contains a set of mandatory shared (core) attributes that should be included regardless of the chosen package. Model-specific attributes are optionally selected by the user. This library is a file in open Excel format, allowing researchers to easily add new standards, update and extend existing standards, and change the preset status of optional and mandatory attributes.

Open-format Excel files can be handled by (open-source) office productivity software on many devices, which opens the way for on-site metadata registration, for instance, while taking a sample. Many attributes have restricted values. Boolean attributes, for instance, are either “true” or “false.” Other values are invalid, and using them compromises structural interoperability and therefore the machine-actionability of the metadata field. At any time during the metadata registration process, the format of restricted metadata values can be checked by simply uploading the Excel workbook to the FAIR-DS.

Restricted values are validated using regular expressions directly obtained from the ENA checklists, such as “(0|((0.)|([1-9][0-9]*.?))[0-9]*)([Ee][+-]?[0-9]+)? (g|mL|mg|ng)” for sample volume or weight for DNA extraction [12]. In addition, the FAIR-DS can validate user-recorded ontology terms. When an URL of the corresponding OWL file is provided, the OWL file is automatically retrieved and transformed into an RDF database. During the validation process, user-recorded ontology terms are checked against rdfs:label values of the corresponding ontology. As a working example, we have implemented ontology term validation of the Environment Ontology obtained from http://purl.obolibrary.org/obo/envo.owl). Regular expressions and URLs are stored in the external metadata library file. This file can be exported as ELIXIR Biovalidator JSON Schema files [13].

Other checks include activation of unsolicited auto-complete and auto-correction (Excel) functions, such as the transformation of a numeric value to a calendar date, and for mismatches between identifiers used at the different ISA levels.

## Querying Metadata

Having your experiment metadata at hand in a machine-actionable format is key for efficient downstream data analysis. After validation, the Excel workbook is automatically exported as a Resource Description Framework (RDF) document in Turtle format. Multiple ontologies and terms are incorporated (FOAF, JERM, PPEO, Linked-ISA, PROV, Schema.org, and MIxS) [10, 14–19] to generate an understandable resource of the experiment metadata. Overlapping ISA terms are linked using equivalent to mapping. This document can be directly ingested in a triple store, thereby creating the opportunity for researchers to query their metadata from different programming languages such as R, Python, or Java and to incorporate the metadata in their analysis workflows.

The impact of such a resource will become even more significant if the FAIR-DS is used for gathering metadata of multiple research projects revolving around a common theme. Bringing together multiple project-specific metadata RDF documents enables crosswalks between similar projects, which allows for questions such as “retrieve the ID of all samples for which attribute X is true.” Without a proper metadata management system, such simple questions would be nearly impossible to ask.

In addition, we use these RDF documents to automate downstream data analysis processes such as computational workflows and to support data infrastructures.

## ENA Submission of Sequence Files

One of the public resources for sharing and publishing nucleotide data is the ENA as part of the ELIXIR infrastructure [20]. To convert research metadata into an ENA-acceptable format, an ENA submission module is implemented as an extension of the Resource module. This module accepts a validated RDF metadata file as input and converts Study, Observation unit, Sample, and Assay metadata into ENA-compatible XML files that can be directly uploaded to the ENA submission portal. ENA accession PRJEB54921 describing amplicon sequencing data and PRJEB56403 and PRJEB58924 [21] describing genome sequence data are examples of such an ENA submission.

## Implementation and Documentation

The FAIR-DS is a web-based Java application using Vaadin as a front end [22]. It is available as a JAR package and as a Docker image and can be executed out of the box without additional dependencies as a private or local instance. The FAIR-DS supports the FAIR-by-Design principles that aim to collect FAIR experiment metadata already from the first phase of a project.

Documentation is available via https://docs.fairbydesign.nl and from within the application. This includes technical information on how to set up the FAIR-DS, how to modify and extend an existing metadata model, and how to add a new model. For users, it is explained with telling examples in detail how to register and validate metadata, how to query the validated and converted data files, and how to create a sequence-related metadata XML file for submission to ENA.

## Conclusions

The FAIR-DS is a lightweight stand-alone application for metadata management and validation and was developed as an integral part for the UNLOCK infrastructure (https://m-unlock.nl) for exploring new horizons for research on microbial communities [23]. It has multiple features that enhance usability and interoperability: first, portability, the FAIR-DS can be used as a stand-alone Java application, including all dependencies. No additional installation steps are needed to use this program. Second is the usage of Excel Workbooks in open Excel format as a familiar environment for metadata registration. Out-of-the-box Excel Workbooks provide multiple ways to present a clear overview of the metadata and enable cooperation and offline management. The use of Excel Workbooks for sample registration separates the FAIR-DS from Dendro, CEDAR, *-DCC, and COPO as these FAIRification tools are fully web based [24–27]. Last, the ability to automatically generate machine-actionable ENA metadata submission files will ease the hassles of creating such high-quality metadata and will increase the FAIRness of sequence data submissions.

## Availability of Source Code and Requirements

Project name: FAIR Data Station

Project homepage: https://fairbydesign.nl

Project code repository: https://gitlab.com/m-unlock/fairds

Documentation: https://docs.fairbydesign.nl

Operating system(s): Platform independent

Programming language: Java

Other requirements: Java 11 or higher

License: Apache License 2.0

RRID:SCR_023239

BioTools: biotools:fair_data_station

## Data Availability

An archival copy of the code is also available via the *GigaScience* repository, GigaDB [28].

## Abbreviations

ENA: European Nucleotide Archive; FAIR: Findable, Accessible, Interoperable, Reusable; FAIR-DS: FAIR Data Station; ISA: Investigation, Study, and Assay; MIxS: minimum information standard checklists about any (x) Sequence; RDF: Resource Description Framework.

## Competing Interests

The authors declare that they have no competing interests.

## Funding

B.N., P.J.S., and J.J.K. acknowledge the Dutch national funding agency NWO and Wageningen University and Research for their financial contribution to the UNLOCK initiative (NWO: 184.035.007).

## Supplementary Material

giad014_GIGA-D-22-00282_Original_Submission

giad014_GIGA-D-22-00282_Revision_1

giad014_GIGA-D-22-00282_Revision_2

giad014_GIGA-D-22-00282_Revision_3

giad014_Response_to_Reviewer_Comments_Original_Submission

giad014_Response_to_Reviewer_Comments_Revision_1

giad014_Response_to_Reviewer_Comments_Revision_2

giad014_Reviewer_1_Report_Original_Submission Dominique Batista -- 11/13/2022 Reviewed

giad014_Reviewer_2_Report_Original_Submission Sveinung Gundersen, Ph. D. -- 11/16/2022 Reviewed

giad014_Reviewer_2_Report_Revision_1 Sveinung Gundersen, Ph. D. -- 1/30/2023 Reviewed
