# Supplementary material for: FAIR data station for lightweight metadata management and validation of omics studies
Source: Gigascience. 2023 Mar 6;12:giad014. doi: 10.1093/gigascience/giad014 (PMC9989329; doi:10.1093/gigascience/giad014)
Supplement: giad014_GIGA-D-22-00282_Revision_1 [file giad014_giga-d-22-00282_revision_1.pdf]

GigaScience

FAIR Data Station for Lightweight Metadata Management & Validation of Omics Studies

--Manuscript Draft--

**Manuscript Number:**	GIGA-D-22-00282R1
**Full Title:**	FAIR Data Station for Lightweight Metadata Management & Validation of Omics Studies
**Article Type:**	Technical Note
**Funding Information:**	NWO (184.035.007) — Msc Bart Nijsse<br>Dr Peter Schaap<br>Dr Jasper Jan Koehorst
**Abstract:**	Background: The Life sciences are one of the the biggest suppliers of scientific data. Reusing and connecting this data can uncover hidden insights and lead to new concepts. Efficient reuse of these data sets is strongly promoted when they are interlinked with a sufficient amount of machine-actionable metadata. While the FAIR guiding principles have been accepted by all stakeholders, in practice there are only a limited number of easy to adopt implementations available that fulfil the needs of data producers.<br>Findings: We developed the FAIR Data Station, a lightweight application written in Java, that aims to support researchers in managing research metadata according to the FAIR principles. It implements the ISA metadata framework and uses minimal information metadata standards to capture experiment metadata. The FAIR Data Station consists of three modules. Based on the minimal information model(s) selected by the user, the “form generation module” creates a metadata template Excel workbook with a header row of machine actionable attribute names. The Excel workbook is subsequently used by the data producer(s) as a familiar environment for sample metadata registration. At any point during this process the format of the recorded values can be checked using the “validation module”. Finally, the “resource module” can be used to convert the set of metadata recorded in the Excel workbook in RDF format, enabling (cross-project) (meta)data searches and, for publishing of sequence data, in an European Nucleotide Archive compatible XML metadata file.<br>Conclusions: Turning FAIR into reality requires the availability of easy to adopt data FAIRification workflows that are also of direct use for data producers. As such the FAIR Data Station provides in addition to the means to correctly FAIRify (Omics) data, the means to build searchable metadata databases of similar projects and can assist in ENA metadata submission of sequence data. The FAIR Data Station is available at https://fairbydesign.nl.
**Corresponding Author:**	Jasper Jan Koehorst<br>Wageningen University & Research<br>Wageningen, NETHERLANDS
**Corresponding Author Secondary Information:**	
**Corresponding Author's Institution:**	Wageningen University & Research
**Corresponding Author's Secondary Institution:**	
**First Author:**	Bart Nijsse
**First Author Secondary Information:**	
**Order of Authors:**	Bart Nijsse
	Peter Schaap
	Jasper Jan Koehorst
**Order of Authors Secondary Information:**	

Powered by Editorial Manager® and ProduXion Manager® from Aries Systems Corporation

Response to Reviewers:	Dear Dr. Zauner,<br>We are grateful for the editorial and referral critique of our paper entitled "FAIR Data Station for Lightweight Metadata Management & Validation of Omics Studies" (GIGA-D-22-00282) that was submitted to GigaScience. The reviewers’ comments led to further improvements of the FAIR Data Station and associated documentation. We have also modified the manuscript to further clarify the points raised by the reviewers.<br>Reviewer reports:<br><br>We thank the reviewers for bringing to our attention several issues that needed to be resolved in order for the ap plication to be more suitable and clear to the reader. We did our utmost to address all comments, and believe that thanks to this “FAIR Data Station for Lightweight Metadata Management & Validation of Omics Studies” is a better manuscript than it was prior to revision.<br><br>Reviewer #1: An overall a strong paper that creates a new bridge between the ISA model and the FAIR principles.<br>A few points should be addressed:<br><br>- page 2:<br>* "As one Investigation can have several research lines, each Study layer has a unique identifier ...": how do you generate these identifiers and control their uniqueness, persistency and stability? Are these identifiers resolvable ?<br><br>Response:<br>Study identifiers are created by the researchers and must be between 5 and 25 characters by default. Duplicated identifiers are not allowed within a single investigation and will be flagged during the validation process. Note that the FAIR-DS is a metadata validator for local usage. It is up to the users to use resolvable identifiers.<br><br>* "As an extension to the original three-tier ISA-model in between Study and Assay two additional layers of information were added Observation unit and Sample": would you clarify what problems were addressed? More generally speaking, does the FAIR-DS integrate with existing implementation of the ISA model? Did you consider a conversion and submission to external systems such as the ones mentioned in the conclusion?<br><br>Response:<br>As an extension to the original three-tier ISA-model in between Study and Assay two additional layers of information were added. We noticed that the experimentalists/data producers are confused by the terms “source material” and “sample material” as for them they are too similar. We therefore implemented the object types "Observation unit", described in the ISA-Tab format for MIAPPE v1.1 (http://miappe.org) as a replacement for “source material” and implemented the more familiar “Sample” from the Just Enough Results Model (http://jermontology.org). For experimentalists / data producers this extension make sense as the minimal information models applied, focus on contextual data of the sampling environment.<br><br>In addition, we have investigated different ISA ontologies available being the (i) Just Enough Results model (JERM, 2013), (ii) LinkedISA (2014) of which the terms were not resolvable and (iii) a new unreleased version using WikiData terms, ISA-JSONLD (2021). Due to observed discrepancies, we mainly focused on the stable JERM version.<br><br>We agree with the reviewer that by focussing on JERM we have deviated from the original ISA model. After discussion with other ISA users and with ISA developers we have modified the schema used in the FAIR Data Station to include the latest version of the RDF ontology using the concepts exemplified here: https://github.com/ISA-tools/isa-api/blob/master/isa-cookbook/content/notebooks/ISA-jsonld-basic-test.ttl and mapped “Observation unit” to “source material” and Sample to “sample material”.<br><br>We have updated the manuscript accordingly. In the main text we now mention that the “schema is aligned with the current ISA model by linking "Observation unit" and “Sample” to the equivalent classes “source material” and “sample material” respectively.”

Powered by Editorial Manager® and ProduXion Manager® from Aries Systems Corporation

An example is available in our git repository (https://gitlab.com/m-unlock/fairds under the examples section).

Did you consider a conversion and submission to external systems such as the ones mentioned in the conclusion?

Response:
Yes, experimentalists often experience the process of data FAIRification as a burden. We therefore think it is important to give them something in return that can be used immediately: a simple procedure to get an ENA accession number for sequence data. As indicated in the main text under ENA submission of sequence files the FAIR-DS is able to automatically generate the required metadata input files. A small list of successful (metadata) submissions amplicon data and genome data are mentioned in the main text. See for instance https://www.ebi.ac.uk/ena/browser/view/PRJEB56403 describing a genome sequence data set. Advanced users can export the FAIR-DS metadata library in a JSON format which makes it possible to use the ELIXIR Biovalidator: https://doi.org/10.1093/bioinformatics/btac195). In the main text we have now made a referral to this option.

* The text for figure 1 is good, but the corresponding text in the core of the document is hard to read and understand.

We have reviewed the corresponding text in the main document

* "Model specific attributes are optionally selected by the user": Does this mean users can add extra fields on top of the provided packages or that they have to select fields within the given package?

Response:

Each chosen package represents a community accepted minimal information model for a particular type of study and is presented to the user as a complete list with mandatory and optional fields. In addition, other (custom) fields can be manually added to Excel workbook. By selecting “Term Overview” in the menu, the FAIR-DS tool provides a complete overview of available field–value pairs including example values. From a FAIR point of view, it is advantageous to select additional fields from this list as these are interoperable and restricted field formats will be automatically included in the validation process. Other custom fields can be added by simply adding a custom column header in the appropriate excel sheet. As custom field–value pairs are not registered in the FAIR-DS library, they will be ignored in the validation process. However, as they will be present in the exported Turtle file, these field–value pairs can still be queried and used for post processing. Note that we have developed a simple procedure to add new field–value pairs and value specifications to existing packages. (See also below)

-page 3:
* "In addition, we included regular expressions obtained from the ENA checklist, such as "(0|((0˙)|([1-9][0-9]*˙?))[0-9]*)([Ee][+-]?[0-9]+)? (g|mL|mg|ng)" for sample volume or weight for DNA extraction": good point. Is their a mechanism for users to add new regex ?

Response:
Field–value pairs, value specifications, examples and regular expressions are stored in an external metadata library file in an open excel format. 1) When users use their own local instance of the FAIR Data Station, this template file can be modified to add new or update existing regular expressions, to add new fields and to add or replace packages. 2) As the FAIR-DS is an integrated part of the Unlock large scale infrastructure (https://m-unlock.nl), newer versions of this metadata template file will be made available along with new releases of the FAIR-DS.

Reviewer #2: The paper describes the FAIR Data Station, which is a lightweight application written in Java that facilitates FAIR-by-design by allowing the collection of structured metadata from the first phase of a project. To this end, the authors have

Powered by Editorial Manager® and ProduXion Manager® from Aries Systems Corporation

applied and extended the ISA metadata framework to form a core data structure wherein attributes from a library of 40 frequently used minimal information checklists can be placed. The FAIR Data Station contains tools for generating and validating Excel metadata files, as well as conversion to RDF format as well as to a European Nucleotide Archive(ENA) compatible XML metadata file for submission.

General comments:

The FAIR Data Station (FAIR-DS) seems to be a useful application to help life science researchers to collect and structure metadata according to the FAIR principles. The software is based on core community standards, ontologies and checklists. As for deposition databases, the software currently seems to only integrate with ENA, which, on the other hand, is a central deposition database.

The three main contributions of FAIR-DS is to my mind A) the metadata schema that has been carefully constructed by the authors, B) the validation functionality of metadata against said schema, and C) functionality for conversion of validated metadata into RDF and deposition formats

There are, however, some architectural choices and technical limitations in the implementation that I have issues with and which makes me uncertain whether the software shows enough "innovation in the approach, implementation, or have added benefits", as mentioned in the "Instructions for Authors"(https://academic.oup.com/gigascience/pages/technical_note). I would therefore invite the authors to address the following issues:

1. The authors state that "the FAIR-DS uses an extended version of the original three-tier Investigation, Study, Assay (ISA) metadata framework [https://isa-tools.org]". This leads the reader to think that the software applies the full ISA Abstract Model (https://isa-specs.readthedocs.io/en/latest/isamodel.html), which is not correct. Only the top level objects and a few attributes are retained. It is also not clear why the authors have found it necessary to add additional, custom object types, such as "Observation unit", explained as "the "object" from which the measurements are taken". The ISA model includes an attribute "source material" which seems to overlap. The authors have also added "sample" as a top-level object, even though there is already a "sample" attribute in the ISA model. It is unclear to me what is improved by adding new object types and whether any such improvements will outweigh the obvious drawbacks that comes with not following a community standard for the metadata schema.

Response:

The reviewer is correct that at some points we deviated from the ISA standard. As indicated above, during the development of the tool, we noted that for most experimentalists/data producers “source material” and “sample material” refer to the same thing. We therefore implemented the object types "Observation unit", used in the MIAPPE v1.1 ISA-Tab format (https://github.com/MIAPPE/ISA-Tab-for-plant-phenotyping) and Sample from the Just Enough Results Model (https://jermontology.org). From the perception of an experimentalist / data producer this separation make sense as the minimal information models used, focus on static and dynamic contextual data of the sampling environment.
After discussion with other ISA users and developers we have aligned the schema used in the FAIR-DS with the current example of the RDF dataset shown here: https://github.com/ISA-tools/isa-api/blob/master/isa-cookbook/content/notebooks/ISA-jsonld-basic-test.ttl and mapped “Observation unit” and “Sample” to source and sample material (https://gitlab.com/m-unlock/fairds under the examples section).

We have updated the manuscript accordingly. In the main text we now mention that the “schema is aligned with the current ISA model by linking "Observation unit" and “Sample” to the equivalent classes “source material” and “sample material” respectively.”

2. The FAIR-DS makes use of Excel files as an intermediate format for collection of

Powered by Editorial Manager® and ProduXion Manager® from Aries Systems Corporation

user metadata. While the feature set of Excel and its familiarity for most users are good arguments its adoption, I miss a discussion on the fact that a commercial product is included in the core architecture of the system. FAIR principle I1 promote that: "(Meta)data use a formal, accessible, shared, and broadly applicable language for knowledge representation". As Excel is only an intermediate metadata format, while RDF is used for the final output, the FAIR-DS does not directly break principle I1, however I think the choice of a commercial file format is not following the "spirit" of FAIR. I see no reason why CSV could not be included as an alternative to Excel and that the authors could recommend an Open Source application as alternative for users that wish their entire software suite to remain in the Open Source domain.

Response:

To encourage researchers to FAIRify their data we have to find a compromise between the work imposed on the data producer and the ideal world of the data consumers. The required FAIRification tasks must be clear and useful, yet not be of a burden to compile, that submitters would avoid metadata entry. As this tool is developed as a practical implementation for experimentalists/data producers there are concessions. The current situation is that for security reasons experimentalists are not always allowed to manage their own workstation and therefore depend on a standard collection of productivity software including Excel. In the spirit of FAIR, we use the Open XML format for the Excel files. What is important here is that Excel is not used for data transformation. While it is true that Excel is more explicitly mentioned however, alternative open spreadsheet software such as open office and libre office are used internally and are working as expected.

In the manuscript we have now put more emphasis on the use of the open Excel format

With regards to the CSV formatting, within the metadata workbook there are multiple sheets representing the different ISA levels bundled in, again from an experimental point of view, logical order (Observation Unit, Sample, Assay). Experimentalists/data producers therefore can directly use the metadata captured in the Excel workbook for their own data analysis while the RDF output is available for more advanced users. It is indeed technically very well possible to split this into separate CSV files, but this brings an additional level of complexity to the user. After discussion with multiple (experienced) experimentalists/data producers it became apparent that they would use spreadsheet software to generate the required CSV files.

3. The metadata schema is not represented in a standard schema format, such as JSON Schema, Frictionless table schema, or similar. Using a shared format for representing the metadata schema makes it possible to make use of general validation libraries (such as the ELIXIR Biovalidator: https://doi.org/10.1093/bioinformatics/btac195). Shared schema formats also allows for reuse of the schema in other contexts/software. In FAIR-DS, the metadata schema seems to be primarily represented in an implicit way in the Java source code that generates the Excel files as a secondary representation of the schema. Even though the FAIR principles might not directly include a recommendation to share of the metadata schema in a FAIR way, one can argue that this falls under R1.3: "(Meta)data meet domain-relevant community standards". It would in any case be in "the spirit of FAIR".

Response:
For advanced users the FAIR-DS can export the FAIR-DS metadata file into a multitude of JSON files (one for each package) which makes it possible to use the ELIXIR Biovalidator: https://doi.org/10.1093/bioinformatics/btac195). In the main text we have now made a referral to this option

4. As a consequence of issue 3, the validation functionality is also specified implicitly in the Java source code and does not seem to reuse much external validation functionality. I particularly miss validation of ontology terms against the relevant ontologies, as well as more stringent validation of PMIDs, DOIs etc, preferable using CURIEs instead of URLs. All of these data types only seem to be validated as general

strings, which is of limited use. Users might for instance introduce spelling variants for ontology term labels without this being detected by the validator.

Response:

As indicated above this tool is developed as a practical implementation for experimentalists/data producers and inevitably there are some concessions. The checklists are directly obtained from https://www.ebi.ac.uk/ena/browser/checklists and the ENA supplied regular expressions are used when the field format is restricted. We have improved the FAIR-DS metadata model by adding a regular expression for the Document Object Identifier obtained from https://registry.identifiers.org/registry/doi. Although we agree with the reviewer that regular expressions are not always fool proof, together with an included example, they provide a simple and understandable feedback mechanism. Moreover, we think that in practice most users will simply use copy/paste to add fields with more complex restrictions such as for example a DOI.

We are currently exploring the validation of ontology terms. The FAIR-DS metadata file (metadata.xlsx) regular expression field now also accepts an URL-link to an OWL-file. When present the file is retrieved and transformed into an RDF database. During the validation process the user-recorded ontology terms are checked against rdfs:label values of the selected ontology. As a working example we have implemented ontology term validation of the Environment Ontology (http://purl.obolibrary.org/obo/envo.owl).

We have updatedthe main text accordingly

5. Due to the hard-coded nature of the metadata schema, the validator and the conversion functionality, I suspect the authors might not have designed the system flexibly enough to allow for easy updates based on updates in the external dependencies, i.e. the minimal information checklists, ontologies, or deposition schemas. For instance, EMBL-EBI, who are hosting ENA, are moving towards requiring the submission of sample data/metadata to BioSamples, prior to submitting the metadata to ENA, which might have consequences for the checklist requirements. Also, ontologies in particular are known to be updated regularly.

Response:
The checklists are directly obtained from https://www.ebi.ac.uk/ena/browser/checklists where they are presented in a simple tabular format. The current ENA download format at this official site is in XML. For practical reasons these XML files were converted to Excel and merged in a single FAIR-DS metadata file. Upon start the application will generate in a subfolder a minimal information model checklist file in xlsx format (metadata.xlsx) that is used as an internal database. This self-explanatory file, in strict open (xlsx) format, functions as the checklists “database”. We have chosen for this solution as it provides users with limited digital skills that want to run a local instance of the FAIR-DS with a simple mechanism to make optional fields mandatory and to add new or update existing packages. As indicated above, when required, the FAIR-DS tool can export this database in JSON format using the FAIR Data Station. Furthermore, we use the tool to submit sequence data to ENA. Procedural changes implemented by the EMBL-EBI, will therefore result in a new release of the FAIR Data Station metadata.

6. I am not convinced that the authors have done a careful enough search of the literature to list relevant software solutions for comparison. For instance, the FAIRDOM Seek solution (https://doi.org/10.1186/s12918-015-0174-y) is not cited directly, although the functionality seems to be highly overlapping.

Response:
Thank you for mentioning the SEEK platform. We are currently hosting a SEEK system in our infrastructure however from experience in interaction with experimentalists / data producers we have noted that this platform is not suitable for routine recording of metadata when many samples are involved. We are in close collaboration with the developers of SEEK (Manchester) to see if such integration is possible in the (near) future.

7. The manuscript would benefit from careful proofreading of the language and grammar.

	When addressing these issues, I would urge the authors to better demonstrate "innovation in the approach, implementation, or ... added benefits",<br><br>Response:<br>We have carefully corrected the manuscript to improve the language and grammar style.
Additional Information:	
Question	Response
Are you submitting this manuscript to a special series or article collection?	No
**Experimental design and statistics**<br><br>Full details of the experimental design and statistical methods used should be given in the Methods section, as detailed in our Minimum Standards Reporting Checklist. Information essential to interpreting the data presented should be made available in the figure legends.<br><br>Have you included all the information requested in your manuscript?	No
If not, please give reasons for any omissions below.<br><br>as follow-up to "**Experimental design and statistics**<br><br>Full details of the experimental design and statistical methods used should be given in the Methods section, as detailed in our Minimum Standards Reporting Checklist. Information essential to interpreting the data presented should be made available in the figure legends.<br><br>Have you included all the information requested in your manuscript?<br><br>"	Not applicable for this paper.
**Resources**<br><br>A description of all resources used,	Yes

including antibodies, cell lines, animals and software tools, with enough information to allow them to be uniquely identified, should be included in the Methods section. Authors are strongly encouraged to cite Research Resource Identifiers (RRIDs) for antibodies, model organisms and tools, where possible.<br><br>Have you included the information requested as detailed in our Minimum Standards Reporting Checklist?	
**Availability of data and materials**<br><br>All datasets and code on which the conclusions of the paper rely must be either included in your submission or deposited in publicly available repositories (where available and ethically appropriate), referencing such data using a unique identifier in the references and in the "Availability of Data and Materials" section of your manuscript.<br><br>Have you have met the above requirement as detailed in our Minimum Standards Reporting Checklist?	Yes

Powered by Editorial Manager® and ProduXion Manager® from Aries Systems Corporation

Click here to access/download;Manuscript;FAIR_DATA_STATION-8.pdf

GigaScience, 2022, 1–4

doi: xx.xxxx/xxxx

PAPER

FAIR Data Station for Lightweight Metadata Management & Validation of Omics Studies

Bart Nijsse[1,2], Peter J. Schaap[1,2] and Jasper J. Koehorst[1,2,*]

[1]Laboratory of Systems and Synthetic Biology, Wageningen University & Research, Wageningen, The Netherlands and [2]UNLOCK Large Scale Infrastructure for Microbial Communities, Wageningen University & Research and Delft University of Technology, The Netherlands

[*] Corresponding author jasper.koehorst@wur.nl

Abstract

Background: The Life sciences are one of the the biggest suppliers of scientific data. Reusing and connecting this data can uncover hidden insights and lead to new concepts. Efficient reuse of these data sets is strongly promoted when they are interlinked with a sufficient amount of machine-actionable metadata. While the FAIR guiding principles have been accepted by all stakeholders, in practice there are only a limited number of easy to adopt implementations available that fulfil the needs of data producers.
Findings: We developed the FAIR Data Station, a lightweight application written in Java, that aims to support researchers in managing research metadata according to the FAIR principles. It implements the ISA metadata framework and uses minimal information metadata standards to capture experiment metadata. The FAIR Data Station consists of three modules. Based on the minimal information model(s) selected by the user, the "form generation module" creates a metadata template Excel workbook with a header row of machine actionable attribute names. The Excel workbook is subsequently used by the data producer(s) as a familiar environment for sample metadata registration. At any point during this process the format of the recorded values can be checked using the "validation module". Finally, the "resource module" can be used to convert the set of metadata recorded in the Excel workbook in RDF format, enabling (cross-project) (meta)data searches and, for publishing of sequence data, in an European Nucleotide Archive compatible XML metadata file.
Conclusions: Turning FAIR into reality requires the availability of easy to adopt data FAIRification workflows that are also of direct use for data producers. As such the FAIR Data Station provides in addition to the means to correctly FAIRify (Omics) data, the means to build searchable metadata databases of similar projects and can assist in ENA metadata submission of sequence data. The FAIR Data Station is available at `https://fairbydesign.nl`.

Key words: FAIR; Metadata; MIxS standards; ENA submission tool; Semantic Web; Ontologies;

Background

Online repositories sharing scientific data are vital for the advancement of science. Data sharing improves research transparency, promotes the validation of experimental methods and scientific conclusions, enables data reuse and facilitates knowledge discovery using new analysis tools. Essential for reusing scientific data is the availability of machine-readable metadata about the scientific experiments conducted with a degree of completeness that reflects the FAIR guiding principles: Findable, Accessible, Interoperable, Reusable [1].

Several concepts have been developed to assist in the data FAIRification process. The ISA metadata framework standard [2] specifies an abstract model to capture experiment metadata using three core levels, Investigation, Study and Assay. The GO-FAIR initiative [3] suggests a seven-step workflow for data FAIRification. They, however, do not provide practical implementations of the technological components needed in the FAIRification process. This is because FAIR is not a standard, but a set of guidelines open to interpretation.

A key feature of properly FAIRified data is a high level of data Interoperability. From a data producer/user point of view two levels are important: structural and semantic interoperability. Structural interoperability defines the format of the data, allowing the

Compiled on: January 19, 2023.

data to be interpreted by multiple systems. For example, the FASTA sequence format is the most implemented and best machine-actionable data standard for sequence data data and therefore directly understood by many sequence analysis tools [4, 5]. Semantic interoperability entails the transformation of ambiguous human-understandable metadata in a standardized machine-actionable open format, allowing computational support systems to automatically find, access, and reuse data. To ensure that the set of metadata is sufficient for the data to be unambiguously described, standardized minimal information models and checklists, detailing those requirements, have been developed for wide array of experiment data [6].

Next generation high-throughput sequencing experiments are the major big data generators of the Life Sciences [7]. Sequence data is a special case as it implies a large-scale assessment of a single type of molecules. This property and its representation in standard FASTA format make the sequence data type an excellent candidate for data reuse. To assist in the FAIRification process of sequence data, the Genomic Standards Consortium [8] has developed a widely accepted family of minimum information standard checklists about any (x) Sequence (MIxS). While these guidelines were developed with sequence data in mind, they can also be used to describe sample metadata of other studies.

To help researchers to FAIRify their experiment data in line with accepted standards we have developed the FAIR Data Station (FAIR-DS). The overall goal of this lightweight stand-alone tool is to assist the domain researcher / data producer in creating high-quality FAIR metadata. The FAIR-DS supports the MIxS set of metadata standards implemented by the main sequence databases such as the European Nucleotide Archive (ENA), Genbank, MGnify (EBI Metagenomics), JGI-GOLD and others (see https://doi.org/10.25504/FAIRsharing.9aa0zp for more) and can be used to streamline metadata submission of sequence data to ENA. The output of the FAIR-DS can also be directly used to build a metadata database of (similar) projects, while the default set of mandatory and optional metadata fields can easily be expanded to align with the internal standards of a research group.

Design considerations

For metadata registration the FAIR-DS uses an amended version of the original three level Investigation, Study, Assay (ISA) metadata framework [https://isa-tools.org]. The Investigation layer contains human readable project related metadata: title, authors and a minimal amount of high-level information to understand the overall goals of the experiment(s). The Study layer describes a specific research line. As one investigation can have several research lines, each Study layer has a unique user-defined identifier, a study title, and a description of the experimental design of the specific line of research.

As an extension to the original ISA-model in between Study and Assay two additional layers of information were added. While developing the tool we noticed that experimentalists/data producers find the terms "source material" and "sample material" confusingly similar. We therefore implemented the object types "Observation unit", described in the ISA-Tab format for MIAPPE v1.1 (http://miappe.org) as a replacement for "source material" and implemented the more familiar "Sample" from the Just Enough Results Model (http://jermontology.org). For experimentalists / data producers adding these two layers make sense as the minimal information models applied, focus on contextual data of the sampling environment. The amended schema is aligned with the current ISA model by linking "Observation unit" and "Sample" to the equivalent classes "source material" and "sample material" respectively.

The number of Observation units used should be in line with the experimental design. The Sample layer describes the conditions

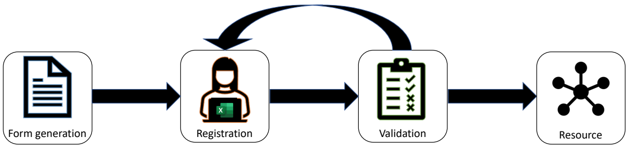

Figure 1. FAIR-Data station metadata registration workflow. The FAIR-Data station workflow consists of three main modules. Based on the minimal information checklist(s) selected by the user the "form generation module" creates a standardised metadata template excel workbook, the "validation module" checks the format-restricted metadata recorded in the workbook. The "resource module" exports the complete set of recorded metadata into an RDF data file, enabling (cross-project) metadata searches, and optionally into ENA compatible metadata submission files.

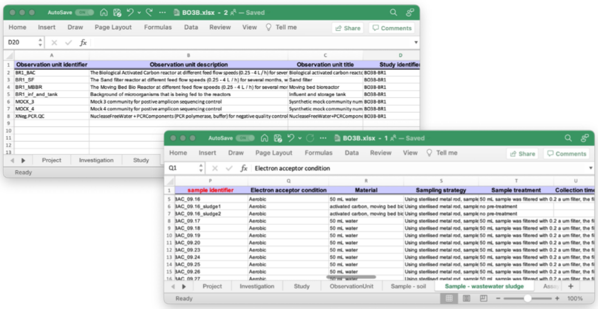

Figure 2. Snapshots of a project metadata workbook generated by the FAIR data station showing the Observation Unit and Sample worksheets. Column headers represent the mandatory and optional attributes (including instruction notes) selected by the user. Each line represents the metadata values associated with a single observation unit or sample. While the columns are in a default order, they can be rearranged to user's preference and user-defined (comment) columns such as in this example "Electron acceptor condition" can be added. User-defined attribute-value pairs are not validated but user-defined column headers will be used as predicates in the RDF knowledge graph. Note that this a multi-sheet workbook in accordance with the ISA standard.

under which a biological sample was taken from an Observation unit. A multitude of samples can be taken from a single observational unit. Each of these samples may also be subjected to multiple Assays.

To encourage domain researchers to FAIRify their data in the best possible way, a metadata registration tool should be flexible, and require little or no training. For the human readable high-level metadata registration, we have chosen for an intuitive web form. Next the tool prompts users to choose one or more minimal information model(s) that best represents the type of samples taken (Figure 1). The chosen model(s) specify a set of mandatory and optional attributes that should be used to describe the samples taken. After selection of the most appropriate minimal information model(s) and relevant optional attributes, the FAIR-DS will generate a metadata template workbook in an open Excel format that will allow sample metadata registration in the form of attribute name-value pairs (Figure 2). The open Excel format was chosen because it allows for offline, on-site metadata registration and supports collaborative efforts and information collection in high throughput. To be able to link different sample types to an observation unit and multiple assay types to a sample, multiple minimal information models can be selected in parallel which will become available as individual sheets in the workbook.

Metadata selection and validation

To assist domain researchers in creating high-quality FAIR metadata the FAIR-DS comes with a metadata package library of 40 frequently used minimal information checklist: 23 are MIxS standards [9] not limited to sequence data and 17 minimal information

checklists directly obtained from ENA [10]. Each individual package contains a set of mandatory shared (core) attributes that should be included regardless of the chosen package. Model specific attributes are optionally selected by the user. This library is a file in open Excel format allowing researchers to easily add new standards, update and extend existing standards and change the pre-set status of optional and mandatory attributes.

Open format Excel files can be handled by (open-source) office productivity software on many devices which opens the way for on-site metadata registration, for instance while taking a sample. Many attributes have restricted values. Boolean attributes for instance, are either "true" or "false". Other values are invalid and using them compromises structural interoperability and therefore the machine-actionability of the metadata field. At anytime during metadata registration process, the format of restricted metadata values can be checked by simply uploading the Excel workbook to the FAIR-DS.

Restricted values are validated using regular expressions directly obtained from the ENA checklists, such as “(0|((0)|([1-9][0-9]*?))[0-9]*)([Ee][+-]?[0-9]+)? (g|mL|mg|ng)” for sample volume or weight for DNA extraction. [11]. In addition, the FAIR-DS can validate user-recorded ontology terms. When an URL of the corresponding OWL-file is provided, the OWL file is automatically retrieved and transformed into an RDF database. During the validation process user-recorded ontology terms are checked against rdfs:label values of the corresponding ontology. As a working example we have implemented ontology term validation of the Environment Ontology obtained from (http://purl.obolibrary.org/obo/envo.owl). Regular expressions and URLs are stored in the external metadata library file. This file can be exported as ELIXIR Biovalidator JSON files [12].

Other checks include activation of unsolicited auto-complete and auto-correction (Excel) functions such as the transformation of a numeric value to a calendar date, and for mismatches between identifiers used at the different ISA levels.

Querying metadata

Having your experiment metadata at hand in a machine-actionable format is key for efficient downstream data analysis. After validation the Excel workbook is automatically exported as a Resource Description Framework (RDF) document in Turtle format. Multiple ontologies and terms are incorporated (FOAF, JERM, PPEO, Linked-ISA, PROV, Schema.org and MIxS) [13, 14, 15, 16, 17, 18, 9] to generate an understandable resource of the experiment metadata. Overlapping ISA terms are linked using equivalent to mapping. This document can be directly ingested in a triple store thereby creating the opportunity for researchers to query their metadata from different programming languages such as R, Python or Java and to incorporate the metadata in their analysis workflows.

The impact of such a resource will become even more significant if the FAIR-DS is used for gathering metadata of multiple research projects revolving around a common theme. Bringing together multiple project specific metadata RDF documents enables cross-walks between similar projects, which allows for questions such as “retrieve the ID of all samples for which attribute X is "true". Without a proper metadata management system such simple questions would be nearly impossible to ask.

In addition, we use these RDF documents to automate downstream data analysis processes such as computational workflows and to support data infrastructures.

ENA submission of sequence files

One of the public resources for sharing and publishing nucleotide data is the European Nucleotide Archive as part of the ELIXIR infrastructure [19]. To convert research metadata into an ENA acceptable format, an ENA submission module was implemented as an extension of the Resource module. This module accepts a validated RDF metadata file as input and converts Study, Observation unit, Sample and Assay metadata into ENA compatible XML files that can be directly uploaded to the ENA submission portal. ENA accession PRJEB54921 describing amplicon sequencing data and, PRJEB56403 and PRJEB58924 [20] describing genome sequence data are examples of such an ENA submission.

Implementation and Documentation

The FAIR Data Station (FAIR-DS) is a web-based Java application using Vaadin as a front-end [21]. It is available as a JAR package and as a Docker image and can be executed out-of-the-box without additional dependencies as a private or local instance. The FAIR-DS supports the FAIR-By-Design principles that aims to collect FAIR experiment metadata already from the first phase of a project.

Documentation is available via `https://docs.fairbydesign.nl` and from within the application. This includes technical information on how to set-up the FAIR Data Station, how to modify and extend an existing metadata model and how to add a new model. For users, it is explained with telling examples in detail how to register and validate metadata, how to query the validated and converted data files and how to create sequence related metadata XML file for submission to ENA.

Conclusions

The FAIR Data Station is lightweight stand-alone application for metadata management and validation and was developed as an integral part for the UNLOCK infrastructure [`https://m-unlock.nl`] for exploring new horizons for research on microbial communities [22]. It has multiple features that enhance usability and interoperability: First, portability, the FAIR-DS can be used as a stand-alone Java application including all dependencies. No additional installation steps are needed to use this program. Second is the usage of Excel Workbooks in open Excel format as a familiar environment for metadata registration. Out of the box Excel Workbooks provide multiple ways to present a clear overview of the metadata and enable cooperation and offline management. The use of an Excel Workbooks for sample registration separates the FAIR-DS from Dendro, CEDAR, *-DCC and COPO as these FAIRification tools are fully web-based [23, 24, 25, 26]. Lastly, the ability to automatically generate machine-actionable ENA metadata submission files will ease the hassles of creating such high-quality metadata and will increase the FAIRness of sequence data submissions.

Availability of source code and requirements

- Project name: FAIR Data Station
- Project home page: `https://fairbydesign.nl`
- Project git repository: `https://gitlab.com/m-unlock/fairds`
- Documentation: `https://docs.m-unlock.nl`
- Operating system(s): Platform independent
- Programming language: Java
- Other requirements: Java 11 or higher
- License: Apache License 2.0

Competing Interests

The authors declare that they have no competing interests.

Funding

B.N., P.J.S and J.J.K acknowledge the Dutch national funding agency NWO, and Wageningen University and Research for their financial contribution to the Unlock initiative (NWO: 184.035.007).

References

1. Wilkinson MD, Dumontier M, Aalbersberg IJ, Appleton G, Axton M, Baak A, et al. The FAIR Guiding Principles for scientific data management and stewardship. Scientific data 2016;3(1):1–9.
2. Philippe Rocca-Serra MB Susanna-Assunta Sansone. Specification documentation: ISA-TAB 1.0. Zenodo 2009 1;https://doi.org/10.5281/zenodo.161355#.YufEo_wTtz4.mendeley.
3. Consortium. Go fair initiative: Make your Data & Services Fair. GO FAIR 2020 Jun;http://go-fair.org/.
4. Lipman D, Pearson W. Rapid and sensitive protein similarity searches. Science 1985;227:1435–1441. http://www.sciencemag.org/cgi/doi/10.1126/science.2983426.
5. Zhang H. Overview of sequence data formats. In: Statistical Genomics Springer; 2016.p. 3–17.
6. McQuilton P, Gonzalez-Beltran A, Rocca-Serra P, Thurston M, Lister A, Maguire E, et al. BioSharing: curated and crowd-sourced metadata standards, databases and data policies in the life sciences. Database 2016;2016.
7. Stephens ZD, Lee SY, Faghri F, Campbell RH, Zhai C, Efron MJ, et al. Big data: astronomical or genomical? PLoS biology 2015;13(7):e1002195.
8. Consortium. Genomic standards consortium. Genomic Standards Consortium 2022;http://gensc.org/.
9. Yilmaz P, Kottmann R, Field D, Knight R, Cole JR, Amaral-Zettler L, et al. Minimum information about a marker gene sequence (MIMARKS) and minimum information about any (x) sequence (MIxS) specifications. Nature biotechnology 2011;29(5):415–420.
10. Cummins C, Ahamed A, Aslam R, Burgin J, Devraj R, Edbali O, et al. The European nucleotide archive in 2021. Nucleic Acids Research 2022;50(D1):D106–D110.
11. Amid C, Alako BT, Balavenkataraman Kadhirvelu V, Burdett T, Burgin J, Fan J, et al. The European nucleotide archive in 2019. Nucleic acids research 2020;48(D1):D70–D76.
12. Liyanage I, Burdett T, Droesbeke B, Erdos K, Fernandez R, Gray A, et al. ELIXIR biovalidator for semantic validation of life science metadata. Bioinformatics 2022;38(11):3141–3142.
13. Graves M, Constabaris A, Brickley D. Foaf: Connecting people on the semantic web. Cataloging & classification quarterly 2007;43(3-4):191–202.
14. Wolstencroft K, Owen S, Krebs O, Mueller W, Nguyen Q, Snoep JL, et al. Semantic data and models sharing in systems biology: The just enough results model and the seek platform. In: International Semantic Web Conference Springer; 2013. p. 212–227.
15. Papoutsoglou EA, Faria D, Arend D, Arnaud E, Athanasiadis IN, Chaves I, et al. Enabling reusability of plant phenomic datasets with MIAPPE 1.1. New Phytologist 2020;227(1):260–273.
16. González-Beltrán A, Maguire E, Sansone SA, Rocca-Serra P. linkedISA: semantic representation of ISA-Tab experimental metadata. BMC bioinformatics 2014;15(14):1–15.
17. Lebo T, Sahoo S, McGuinness D, Belhajjame K, Cheney J, Corsar D, et al. Prov-o: The prov ontology. PROV-O 2013;.
18. Guha RV, Brickley D, Macbeth S. Schema. org: evolution of structured data on the web. Communications of the ACM 2016;59(2):44–51.
19. Crosswell LC, Thornton JM. ELIXIR: a distributed infrastructure for European biological data. Trends Biotechnol 2012;30(5):241–2.
20. Azagi T, Dirks RP, Yebra-Pimentel ES, Schaap PJ, Koehorst JJ, Esser HJ, et al. Assembly and Comparison of Ca. Neoehrlichia mikurensis Genomes. Microorganisms 2022;10(6):1134.
21. Consortium. Vaadin: The modern web application platform for Java. Vaadin 2022 Jun;https://vaadin.com.
22. Kleerebezem R, Stouten G, Koehorst J, Langenhoff A, Schaap P, Smidt H. Experimental infrastructure requirements for quantitative research on microbial communities. Current Opinion in Biotechnology 2021;67:158–165.
23. Shaw F, Etuk A, Minotto A, Gonzalez-Beltran A, Johnson D, Rocca-Serra P, et al. COPO: a metadata platform for brokering FAIR data in the life sciences. F1000Research 2020;9(495):495.
24. Rocha da Silva J, Aguiar Castro J, Ribeiro C, Correia Lopes J. Dendro: collaborative research data management built on linked open data. In: European Semantic Web Conference Springer; 2014. p. 483–487.
25. Gonçalves RS, O'Connor MJ, Martínez-Romero M, Egyedi AL, Willrett D, Graybeal J, et al. The CEDAR workbench: an ontology-assisted environment for authoring metadata that describe scientific experiments. In: International Semantic Web Conference Springer; 2017. p. 103–110.
26. Hörtenhuber M, Mukarram AK, Stoiber MH, Brown JB, Daub CO. *-DCC: A platform to collect, annotate, and explore a large variety of sequencing experiments. GigaScience 2020;9(3):giaa024.
