# Supplementary material for: FAIR data station for lightweight metadata management and validation of omics studies
Source: Gigascience. 2023 Mar 6;12:giad014. doi: 10.1093/gigascience/giad014 (PMC9989329; doi:10.1093/gigascience/giad014)
Supplement: giad014_Response_to_Reviewer_Comments_Original_Submission [file giad014_response_to_reviewer_comments_original_submission.pdf]

Author's Response To Reviewer Comments

Close

Dear Dr. Zauner,
We are grateful for the editorial and referral critique of our paper entitled "FAIR Data Station for Lightweight Metadata Management & Validation of Omics Studies" (GIGA-D-22-00282) that was submitted to GigaScience. The reviewers' comments led to further improvements of the FAIR Data Station and associated documentation. We have also modified the manuscript to further clarify the points raised by the reviewers.
Reviewer reports:

We thank the reviewers for bringing to our attention several issues that needed to be resolved in order for the ap plication to be more suitable and clear to the reader. We did our utmost to address all comments, and believe that thanks to this "FAIR Data Station for Lightweight Metadata Management & Validation of Omics Studies" is a better manuscript than it was prior to revision.

Reviewer #1: An overall a strong paper that creates a new bridge between the ISA model and the FAIR principles.
A few points should be addressed:

- page 2:
* "As one Investigation can have several research lines, each Study layer has a unique identifier ...": how do you generate these identifiers and control their uniqueness, persistency and stability? Are these identifiers resolvable ?

Response:
Study identifiers are created by the researchers and must be between 5 and 25 characters by default. Duplicated identifiers are not allowed within a single investigation and will be flagged during the validation process. Note that the FAIR-DS is a metadata validator for local usage. It is up to the users to use resolvable identifiers.

* "As an extension to the original three-tier ISA-model in between Study and Assay two additional layers of information were added Observation unit and Sample": would you clarify what problems were addressed? More generally speaking, does the FAIR-DS integrate with existing implementation of the ISA model? Did you consider a conversion and submission to external systems such as the ones mentioned in the conclusion?

Response:
As an extension to the original three-tier ISA-model in between Study and Assay two additional layers of information were added. We noticed that the experimentalists/data producers are confused by the terms "source material" and "sample material" as for them they are too similar. We therefore implemented the object types "Observation unit", described in the ISA-Tab format for MIAPPE v1.1 (http://miappe.org) as a replacement for "source material" and implemented the more familiar "Sample" from the Just Enough Results Model (http://jermontology.org). For experimentalists / data producers this extension make sense as the minimal information models applied, focus on contextual data of the sampling environment.

In addition, we have investigated different ISA ontologies available being the (i) Just Enough Results model (JERM, 2013), (ii) LinkedISA (2014) of which the terms were not resolvable and (iii) a new unreleased version using WikiData terms, ISA-JSONLD (2021). Due to observed discrepancies, we mainly focused on the stable JERM version.

We agree with the reviewer that by focussing on JERM we have deviated from the original ISA model. After

discussion with other ISA users and with ISA developers we have modified the schema used in the FAIR Data Station to include the latest version of the RDF ontology using the concepts exemplified here: https://github.com/ISA-tools/isa-api/blob/master/isa-cookbook/content/notebooks/ISA-jsonld-basic-test.ttl and mapped "Observation unit" to "source material" and Sample to "sample material".

We have updated the manuscript accordingly. In the main text we now mention that the "schema is aligned with the current ISA model by linking "Observation unit" and "Sample" to the equivalent classes "source material" and "sample material" respectively."

An example is available in our git repository (https://gitlab.com/m-unlock/fairds under the examples section).

Did you consider a conversion and submission to external systems such as the ones mentioned in the conclusion?

Response:
Yes, experimentalists often experience the process of data FAIRification as a burden. We therefore think it is important to give them something in return that can be used immediately: a simple procedure to get an ENA accession number for sequence data. As indicated in the main text under ENA submission of sequence files the FAIR-DS is able to automatically generate the required metadata input files. A small list of successful (metadata) submissions amplicon data and genome data are mentioned in the main text. See for instance https://www.ebi.ac.uk/ena/browser/view/PRJEB56403 describing a genome sequence data set. Advanced users can export the FAIR-DS metadata library in a JSON format which makes it possible to use the ELIXIR Biovalidator: https://doi.org/10.1093/bioinformatics/btac195). In the main text we have now made a referral to this option.

* The text for figure 1 is good, but the corresponding text in the core of the document is hard to read and understand.

We have reviewed the corresponding text in the main document

* "Model specific attributes are optionally selected by the user": Does this mean users can add extra fields on top of the provided packages or that they have to select fields within the given package?

Response:

Each chosen package represents a community accepted minimal information model for a particular type of study and is presented to the user as a complete list with mandatory and optional fields. In addition, other (custom) fields can be manually added to Excel workbook. By selecting "Term Overview" in the menu, the FAIR-DS tool provides a complete overview of available field–value pairs including example values. From a FAIR point of view, it is advantageous to select additional fields from this list as these are interoperable and restricted field formats will be automatically included in the validation process. Other custom fields can be added by simply adding a custom column header in the appropriate excel sheet. As custom field–value pairs are not registered in the FAIR-DS library, they will be ignored in the validation process. However, as they will be present in the exported Turtle file, these field–value pairs can still be queried and used for post processing. Note that we have developed a simple procedure to add new field–value pairs and value specifications to existing packages. (See also below)

-page 3:
* "In addition, we included regular expressions obtained from the ENA checklist, such as "(0|((0˙)|([1-9][0-9]*˙?))[0-9]*)([Ee][+-]?[0-9]+)? (g|mL|mg|ng)" for sample volume or weight for DNA extraction": good point. Is their a mechanism for users to add new regex ?

Response:
Field–value pairs, value specifications, examples and regular expressions are stored in an external metadata library file in an open excel format. 1) When users use their own local instance of the FAIR Data

Station, this template file can be modified to add new or update existing regular expressions, to add new fields and to add or replace packages. 2) As the FAIR-DS is an integrated part of the Unlock large scale infrastructure (https://m-unlock.nl), newer versions of this metadata template file will be made available along with new releases of the FAIR-DS.

Reviewer #2: The paper describes the FAIR Data Station, which is a lightweight application written in Java that facilitates FAIR-by-design by allowing the collection of structured metadata from the first phase of a project. To this end, the authors have applied and extended the ISA metadata framework to form a core data structure wherein attributes from a library of 40 frequently used minimal information checklists can be placed. The FAIR Data Station contains tools for generating and validating Excel metadata files, as well as conversion to RDF format as well as to a European Nucleotide Archive(ENA) compatible XML metadata file for submission.

General comments:

The FAIR Data Station (FAIR-DS) seems to be a useful application to help life science researchers to collect and structure metadata according to the FAIR principles. The software is based on core community standards, ontologies and checklists. As for deposition databases, the software currently seems to only integrate with ENA, which, on the other hand, is a central deposition database.

The three main contributions of FAIR-DS is to my mind A) the metadata schema that has been carefully constructed by the authors, B) the validation functionality of metadata against said schema, and C) functionality for conversion of validated metadata into RDF and deposition formats

There are, however, some architectural choices and technical limitations in the implementation that I have issues with and which makes me uncertain whether the software shows enough "innovation in the approach, implementation, or have added benefits", as mentioned in the "Instructions for Authors"(https://academic.oup.com/gigascience/pages/technical_note). I would therefore invite the authors to address the following issues:

1. The authors state that "the FAIR-DS uses an extended version of the original three-tier Investigation, Study, Assay (ISA) metadata framework [https://isa-tools.org]". This leads the reader to think that the software applies the full ISA Abstract Model (https://isa-specs.readthedocs.io/en/latest/isamodel.html), which is not correct. Only the top level objects and a few attributes are retained. It is also not clear why the authors have found it necessary to add additional, custom object types, such as "Observation unit", explained as "the "object" from which the measurements are taken". The ISA model includes an attribute "source material" which seems to overlap. The authors have also added "sample" as a top-level object, even though there is already a "sample" attribute in the ISA model. It is unclear to me what is improved by adding new object types and whether any such improvements will outweigh the obvious drawbacks that comes with not following a community standard for
the metadata schema.

Response:

The reviewer is correct that at some points we deviated from the ISA standard. As indicated above, during the development of the tool, we noted that for most experimentalists/data producers “source material” and “sample material” refer to the same thing. We therefore implemented the object types "Observation unit", used in the MIAPPE v1.1 ISA-Tab format (https://github.com/MIAPPE/ISA-Tab-for-plant-phenotyping) and Sample from the Just Enough Results Model (https://jermontology.org). From the perception of an experimentalist / data producer this separation make sense as the minimal information models used, focus on static and dynamic contextual data of the sampling environment.
After discussion with other ISA users and developers we have aligned the schema used in the FAIR-DS with the current example of the RDF dataset shown here: https://github.com/ISA-tools/isa-api/blob/master/isa-cookbook/content/notebooks/ISA-jsonld-basic-test.ttl and mapped “Observation unit” and “Sample” to source and sample material (https://gitlab.com/m-unlock/fairds under the examples section).

We have updated the manuscript accordingly. In the main text we now mention that the “schema is

aligned with the current ISA model by linking "Observation unit" and “Sample” to the equivalent classes “source material” and “sample material” respectively.”

2. The FAIR-DS makes use of Excel files as an intermediate format for collection of user metadata. While the feature set of Excel and its familiarity for most users are good arguments its adoption, I miss a discussion on the fact that a commercial product is included in the core architecture of the system. FAIR principle I1 promote that: "(Meta)data use a formal, accessible, shared, and broadly applicable language for knowledge representation". As Excel is only an intermediate metadata format, while RDF is used for the final output, the FAIR-DS does not directly break principle I1, however I think the choice of a commercial file format is not following the "spirit" of FAIR. I see no reason why CSV could not be included as an alternative to Excel and that the authors could recommend an Open Source application as alternative for users that wish their entire software suite to remain in the Open Source domain.

Response:

To encourage researchers to FAIRify their data we have to find a compromise between the work imposed on the data producer and the ideal world of the data consumers. The required FAIRification tasks must be clear and useful, yet not be of a burden to compile, that submitters would avoid metadata entry. As this tool is developed as a practical implementation for experimentalists/data producers there are concessions. The current situation is that for security reasons experimentalists are not always allowed to manage their own workstation and therefore depend on a standard collection of productivity software including Excel. In the spirit of FAIR, we use the Open XML format for the Excel files. What is important here is that Excel is not used for data transformation. While it is true that Excel is more explicitly mentioned however, alternative open spreadsheet software such as open office and libre office are used internally and are working as expected.

In the manuscript we have now put more emphasis on the use of the open Excel format

With regards to the CSV formatting, within the metadata workbook there are multiple sheets representing the different ISA levels bundled in, again from an experimental point of view, logical order (Observation Unit, Sample, Assay). Experimentalists/data producers therefore can directly use the metadata captured in the Excel workbook for their own data analysis while the RDF output is available for more advanced users. It is indeed technically very well possible to split this into separate CSV files, but this brings an additional level of complexity to the user. After discussion with multiple (experienced) experimentalists/data producers it became apparent that they would use spreadsheet software to generate the required CSV files.

3. The metadata schema is not represented in a standard schema format, such as JSON Schema, Frictionless table schema, or similar. Using a shared format for representing the metadata schema makes it possible to make use of general validation libraries (such as the ELIXIR Biovalidator: https://doi.org/10.1093/bioinformatics/btac195). Shared schema formats also allows for reuse of the schema in other contexts/software. In FAIR-DS, the metadata schema seems to be primarily represented in an implicit way in the Java source code that generates the Excel files as a secondary representation of the schema. Even though the FAIR principles might not directly include a recommendation to share of the metadata schema in a FAIR way, one can argue that this falls under R1.3: "(Meta)data meet domain-relevant community standards". It would in any case be in "the spirit of FAIR".

Response:
For advanced users the FAIR-DS can export the FAIR-DS metadata file into a multitude of JSON files (one for each package) which makes it possible to use the ELIXIR Biovalidator: https://doi.org/10.1093/bioinformatics/btac195). In the main text we have now made a referral to this option

4. As a consequence of issue 3, the validation functionality is also specified implicitly in the Java source code and does not seem to reuse much external validation functionality. I particularly miss validation of

ontology terms against the relevant ontologies, as well as more stringent validation of PMIDs, DOIs etc, preferable using CURIEs instead of URLs. All of these data types only seem to be validated as general strings, which is of limited use. Users might for instance introduce spelling variants for ontology term labels without this being detected by the validator.

Response:

As indicated above this tool is developed as a practical implementation for experimentalists/data producers and inevitably there are some concessions. The checklists are directly obtained from https://www.ebi.ac.uk/ena/browser/checklists and the ENA supplied regular expressions are used when the field format is restricted. We have improved the FAIR-DS metadata model by adding a regular expression for the Document Object Identifier obtained from https://registry.identifiers.org/registry/doi. Although we agree with the reviewer that regular expressions are not always fool proof, together with an included example, they provide a simple and understandable feedback mechanism. Moreover, we think that in practice most users will simply use copy/paste to add fields with more complex restrictions such as for example a DOI.

We are currently exploring the validation of ontology terms. The FAIR-DS metadata file (metadata.xlsx) regular expression field now also accepts an URL-link to an OWL-file. When present the file is retrieved and transformed into an RDF database. During the validation process the user-recorded ontology terms are checked against rdfs:label values of the selected ontology. As a working example we have implemented ontology term validation of the Environment Ontology (http://purl.obolibrary.org/obo/envo.owl).

We have updatedthe main text accordingly

5. Due to the hard-coded nature of the metadata schema, the validator and the conversion functionality, I suspect the authors might not have designed the system flexibly enough to allow for easy updates based on updates in the external dependencies, i.e. the minimal information checklists, ontologies, or deposition schemas. For instance, EMBL-EBI, who are hosting ENA, are moving towards requiring the submission of sample data/metadata to BioSamples, prior to submitting the metadata to ENA, which might have consequences for the checklist requirements. Also, ontologies in particular are known to be updated regularly.

Response:
The checklists are directly obtained from https://www.ebi.ac.uk/ena/browser/checklists where they are presented in a simple tabular format. The current ENA download format at this official site is in XML. For practical reasons these XML files were converted to Excel and merged in a single FAIR-DS metadata file. Upon start the application will generate in a subfolder a minimal information model checklist file in xlsx format (metadata.xlsx) that is used as an internal database. This self-explanatory file, in strict open (xlsx) format, functions as the checklists “database”. We have chosen for this solution as it provides users with limited digital skills that want to run a local instance of the FAIR-DS with a simple mechanism to make optional fields mandatory and to add new or update existing packages. As indicated above, when required, the FAIR-DS tool can export this database in JSON format using the FAIR Data Station. Furthermore, we use the tool to submit sequence data to ENA. Procedural changes implemented by the EMBL-EBI, will therefore result in a new release of the FAIR Data Station metadata.

6. I am not convinced that the authors have done a careful enough search of the literature to list relevant software solutions for comparison. For instance, the FAIRDOM Seek solution (https://doi.org/10.1186/s12918-015-0174-y) is not cited directly, although the functionality seems to be highly overlapping.

Response:
Thank you for mentioning the SEEK platform. We are currently hosting a SEEK system in our infrastructure however from experience in interaction with experimentalists / data producers we have noted that this platform is not suitable for routine recording of metadata when many samples are involved. We are in close collaboration with the developers of SEEK (Manchester) to see if such integration is possible in the (near) future.

7. The manuscript would benefit from careful proofreading of the language and grammar.

When addressing these issues, I would urge the authors to better demonstrate "innovation in the approach, implementation, or ... added benefits",

Response:
We have carefully corrected the manuscript to improve the language and grammar style.

Close
