# Supplementary material for: FAIR data station for lightweight metadata management and validation of omics studies
Source: Gigascience. 2023 Mar 6;12:giad014. doi: 10.1093/gigascience/giad014 (PMC9989329; doi:10.1093/gigascience/giad014)
Supplement: giad014_Response_to_Reviewer_Comments_Revision_1 [file giad014_response_to_reviewer_comments_revision_1.pdf]

Author's Response To Reviewer Comments

Close

We have updated the notion of the JSON to JSON Schema and we have amended the paragraph regarding FAIRDOM and it now reads: Several tools have been created to help make data FAIR (Findable, Accessible, Interoperable, and Reusable). The ISA metadata framework standard outlines a model for capturing experiment metadata using three levels: Investigation, Study, and Assay. The FAIRDOM Hub uses the ISA framework to create a collaboration platform for systems biology research, but it does not offer high-throughput validation. The GO-FAIR initiative outlines a 7-step workflow for making data FAIR, but does not include practical implementations for the technology needed. Note that FAIR is not a standard, but a set of guidelines that can be interpreted differently.

Close
