# Supplementary material for: FAIR data station for lightweight metadata management and validation of omics studies
Source: Gigascience. 2023 Mar 6;12:giad014. doi: 10.1093/gigascience/giad014 (PMC9989329; doi:10.1093/gigascience/giad014)
Supplement: giad014_Response_to_Reviewer_Comments_Revision_2 [file giad014_response_to_reviewer_comments_revision_2.pdf]

Author's Response To Reviewer Comments

Close

Dear Hans Zauner,

We have updated the manuscript to include a citation to the gigascience dataset and we have registered the application at BioTools and SciCrunch.

When using the latex template of gigascience in overleaf https://www.overleaf.com/latex/templates/template-for-gigascience-journal-manuscript-submissions/shgtrssvbjhs I am uncertain where to place the orcid'ids. The orcid's mentioned are correct.

All latex files have been added to the submission form including the latest pdf.

Kind regards,

Close
