# Supplementary material for: FAIR data station for lightweight metadata management and validation of omics studies
Source: Gigascience. 2023 Mar 6;12:giad014. doi: 10.1093/gigascience/giad014 (PMC9989329; doi:10.1093/gigascience/giad014)
Supplement: giad014_Reviewer_1_Report_Original_Submission — Dominique Batista -- 11/13/2022 Reviewed [file giad014_reviewer_1_report_original_submission.pdf]

Reviewer Report

Title: FAIR Data Station for Lightweight Metadata Management & Validation of Omics Studies

Version: Original Submission **Date:** 11/13/2022

Reviewer name: Dominique Batista

Reviewer Comments to Author:

An overall a strong paper that creates a new bridge between the ISA model and the FAIR principles.A few points should be addressed:- page 2:* "As one Investigation can have several research lines, each Study layer has a unique identifier ...": how do you generate these identifiers and control their uniqueness, persistency and stability? Are these identifiers resolvable ?* "As an extension to the original three-tier ISA-model in between Study and Assay two additional layers of information were added Observation unit and Sample": would you clarify what problems were addressed ? More generally speaking, does the FAIR-DS integrate with existing implementation of the ISA model ? Did you consider a conversion and submission to external systems such as the ones mentioned in the conclusion ?* The text for figure 1 is good, but the corresponding text in the core of the document is hard to read and understand.* "Model specific attributes are optionally selected by the user": Does this mean users can add extra fields on top of the provided packages or that they have to select fields within the given package ?-page 3:* "In addition, we included regular expressions obtained from the ENA checklist, such as "(0|((0Ë™)|([1-9][0-9]*Ë™?))[0-9]*)([Ee][+-]?[0-9]+)? (g|mL|mg|ng)" for sample volume or weight for DNA extraction": good point. Is their a mechanism for users to add new regex ?

Level of Interest

Please indicate how interesting you found the manuscript: Choose an item.

Quality of Written English

Please indicate the quality of language in the manuscript: Choose an item.

Declaration of Competing Interests

Please complete a declaration of competing interests, considering the following questions:

- Have you in the past five years received reimbursements, fees, funding, or salary from an organisation that may in any way gain or lose financially from the publication of this manuscript, either now or in the future?
- Do you hold any stocks or shares in an organisation that may in any way gain or lose financially from the publication of this manuscript, either now or in the future?
- Do you hold or are you currently applying for any patents relating to the content of the manuscript?

- Have you received reimbursements, fees, funding, or salary from an organization that holds or has applied for patents relating to the content of the manuscript?
- Do you have any other financial competing interests?
- Do you have any non-financial competing interests in relation to this paper?

If you can answer no to all of the above, write 'I declare that I have no competing interests' below. If your reply is yes to any, please give details below.

I declare that I have no competing interests

I agree to the open peer review policy of the journal. I understand that my name will be included on my report to the authors and, if the manuscript is accepted for publication, my named report including any attachments I upload will be posted on the website along with the authors' responses. I agree for my report to be made available under an Open Access Creative Commons CC-BY license (http://creativecommons.org/licenses/by/4.0/). I understand that any comments which I do not wish to be included in my named report can be included as confidential comments to the editors, which will not be published.

Choose an item.

To further support our reviewers, we have joined with Publons, where you can gain additional credit to further highlight your hard work (see: https://publons.com/journal/530/gigascience). On publication of this paper, your review will be automatically added to Publons, you can then choose whether or not to claim your Publons credit. I understand this statement.

Yes Choose an item.
