# Supplementary material for: FAIR data station for lightweight metadata management and validation of omics studies
Source: Gigascience. 2023 Mar 6;12:giad014. doi: 10.1093/gigascience/giad014 (PMC9989329; doi:10.1093/gigascience/giad014)
Supplement: giad014_Reviewer_2_Report_Original_Submission — Sveinung Gundersen, Ph. D. -- 11/16/2022 Reviewed [file giad014_reviewer_2_report_original_submission.pdf]

Reviewer Report

Title: FAIR Data Station for Lightweight Metadata Management & Validation of Omics Studies

Version: Original Submission **Date:** 11/16/2022

Reviewer name: Sveinung Gundersen, Ph. D.

Reviewer Comments to Author:

The paper describes the FAIR Data Station, which is a lightweight application written in Java that facilitates FAIR-by-design by allowing the collection of structured metadata from the first phase of a project. To this end, the authors have applied and extended the ISA metadata framework to form a core data structure wherein attributes from a library of 40 frequently used minimal information checklists can be placed. The FAIR Data Station contains tools for generating and validating Excel metadata files, as well as conversion to RDF format as well as to a European Nucleotide Archive(ENA) compatible XML metadata file for submission.General comments:The FAIR Data Station (FAIR-DS) seems to be a useful application to help life science researchers to collect and structure metadata according to the FAIR principles. The software is based on core community standards, ontologies and checklists. As for deposition databases, the software currently seems to only integrate with ENA, which, on the other hand, is a central deposition database.The three main contributions of FAIR-DS is to my mind A) the metadata schema that has been carefully constructed by the authors, B) the validation functionality of metadata against said schema, and C) functionality for conversion of validated metadata into RDF and deposition formats There are, however, some architectural choices and technical limitations in the implementation that I have issues with and which makes me uncertain whether the software shows enough "innovation in the approach, implementation, or have added benefits", as mentioned in the "Instructions for Authors"(https://academic.oup.com/gigascience/pages/technical_note). I would therefore invite the authors to address the following issues:1. The authors state that "the FAIR-DS uses an extended version of the original three-tier Investigation, Study, Assay (ISA) metadata framework [https://isa-tools.org]". This leads the reader to think that the software applies the full ISA Abstract Model (https://isa-specs.readthedocs.io/en/latest/isamodel.html), which is not correct. Only the top level objects and a few attributes are retained. It is also not clear why the authors have found it necessary to add additional, custom object types, such as "Observation unit", explained as "the "object" from which the measurements are taken". The ISA model includes an attribute "source material" which seems to overlap. The authors have also added "sample" as a top-level object, even though there is already a "sample" attribute in the ISA model. It is unclear to me what is improved by adding new object types and whether any such improvements will outweigh the obvious drawbacks that comes with not following a community standard for the metadata schema.2. The FAIR-DS makes use of Excel files as an intermediate format for collection of user metadata. While the feature set of Excel and its familiarity for most users are good arguments its adoption, I miss a discussion on the fact that a commercial product is included in the core architecture of the system. FAIR principle I1 promote that: "(Meta)data use a formal, accessible, shared, and broadly applicable language for knowledge representation". As Excel is only an intermediate metadata format, while RDF is used for the final output, the FAIR-DS does not

directly break principle I1, however I think the choice of a commercial file format is not following the "spirit" of FAIR. I see no reason why CSV could not be included as an alternative to Excel and that the authors could recommend an Open Source application as alternative for users that wish their entire software suite to remain in the Open Source domain.3. The metadata schema is not represented in a standard schema format, such as JSON Schema, Frictionless table schema, or similar. Using a shared format for representing the metadata schema makes it possible to make use of general validation libraries (such as the ELIXIR Biovalidator: https://doi.org/10.1093/bioinformatics/btac195). Shared schema formats also allows for reuse of the schema in other contexts/software. In FAIR-DS, the metadata schema seems to be primarily represented in an implicit way in the Java source code that generates the Excel files as a secondary representation of the schema. Even though the FAIR principles might not directly include a recommendation to share of the metadata schema in a FAIR way, one can argue that this falls under R1.3: "(Meta)data meet domain-relevant community standards". It would in any case be in "the spirit of FAIR".4. As a consequence of issue 3, the validation functionality is also specified implicitly in the Java source code and does not seem to reuse much external validation functionality. I particularly miss validation of ontology terms against the relevant ontologies, as well as more stringent validation of PMIDs, DOIs etc, preferable using CURIEs instead of URLs. All of these data types only seem to be validated as general strings, which is of limited use. Users might for instance introduce spelling variants for ontology term labels without this being detected by the validator.5. Due to the hard-coded nature of the metadata schema, the validator and the conversion functionality, I suspect the authors might not have designed the system flexibly enough to allow for easy updates based on updates in the external dependencies, i.e. the minimal information checklists, ontologies, or deposition schemas. For instance, EMBL-EBI, who are hosting ENA, are moving towards requiring the submission of sample data/metadata to BioSamples, prior to submitting the metadata to ENA, which might have consequences for the checklist requirements. Also, ontologies in particular are known to be updated regularly.6. I am not convinced that the authors have done a careful enough search of the literature to list relevant software solutions for comparison. For instance, the FAIRDOM Seek solution (https://doi.org/10.1186/s12918-015-0174-y) is not cited directly, although the functionality seems to be highly overlapping.7. The manuscript would benefit from careful proofreading of the language and grammar.When addressing these issues, I would urge the authors to better demonstrate "innovation in the approach, implementation, or ... added benefits",

Level of Interest

Please indicate how interesting you found the manuscript: Choose an item.

Quality of Written English

Please indicate the quality of language in the manuscript: Choose an item.

Declaration of Competing Interests

Please complete a declaration of competing interests, considering the following questions:

- Have you in the past five years received reimbursements, fees, funding, or salary from an organisation that may in any way gain or lose financially from the publication of this manuscript, either now or in the future?
- Do you hold any stocks or shares in an organisation that may in any way gain or lose financially from the publication of this manuscript, either now or in the future?
- Do you hold or are you currently applying for any patents relating to the content of the manuscript?
- Have you received reimbursements, fees, funding, or salary from an organization that holds or has applied for patents relating to the content of the manuscript?
- Do you have any other financial competing interests?
- Do you have any non-financial competing interests in relation to this paper?

If you can answer no to all of the above, write 'I declare that I have no competing interests' below. If your reply is yes to any, please give details below.

I declare that I have no competing interests

I agree to the open peer review policy of the journal. I understand that my name will be included on my report to the authors and, if the manuscript is accepted for publication, my named report including any attachments I upload will be posted on the website along with the authors' responses. I agree for my report to be made available under an Open Access Creative Commons CC-BY license (http://creativecommons.org/licenses/by/4.0/). I understand that any comments which I do not wish to be included in my named report can be included as confidential comments to the editors, which will not be published.

Choose an item.

To further support our reviewers, we have joined with Publons, where you can gain additional credit to further highlight your hard work (see: https://publons.com/journal/530/gigascience). On publication of this paper, your review will be automatically added to Publons, you can then choose whether or not to claim your Publons credit. I understand this statement.

Yes Choose an item.
