# Supplementary material for: FAIR data station for lightweight metadata management and validation of omics studies
Source: Gigascience. 2023 Mar 6;12:giad014. doi: 10.1093/gigascience/giad014 (PMC9989329; doi:10.1093/gigascience/giad014)
Supplement: giad014_Reviewer_2_Report_Revision_1 — Sveinung Gundersen, Ph. D. -- 1/30/2023 Reviewed [file giad014_reviewer_2_report_revision_1.pdf]

Reviewer Report

Title: FAIR Data Station for Lightweight Metadata Management & Validation of Omics Studies

Version: Revision 1 **Date:** 1/30/2023

Reviewer name: Sveinung Gundersen, Ph. D.

Reviewer Comments to Author:

Dear Authors,Thanks for addressing my comments, which included the development of several additional features in the FAIR Data Station. I find that most of my comments has been addressed satisfactory, however there are a few remaining issues I would like to address:2 (only a comment, no change or response needed). The manuscript now explicitly mentions the possibility to use Open Source alternatives to Excel. This has now been tested to work. This is great and addresses my main concern, which was one of practicality. However, the manuscript now refers to the Open XML format of Excel as an "open Excel format", which is a disputed claim (see e.g. https://archive.fosdem.org/2022/schedule/event/lotech_odfbetterthanooxml/), for instance due to the fact that the ISO-certified variant of the file format is not the default one exported from Excel. As Open Source alternatives to Excel do in practice support the Open XML files produced by the FAIR Data station, this is more of a theoretical/implementation issue, especially since this is in any case an intermediate data format. I am thus satisfied with the response in the context of this review. However, I would still urge the developers to confirm (to themselves) whether the OOXML file produced are of the ISO-certified "OOXML Strict" variety and also consider adding support for the fully open ODF file format. As none of these file formats are mentioned by name in the manuscript (as far as I can see), no manuscript changes are in any case necessary.3 (minor review). The authors have implemented functionality for exporting the metadata schema as "ELIXIR Biovalidator JSON files", which is excellent! However, I find this description a bit unclear. The ELIXIR Biovalidator imports the metadata schema in the form of JSON Schema files, only with the addition of a few extra properties to allow for semantic validation (e.g. ontology terms). These extra properties are (I assume) ignored by standard JSON Schema validators, making such JSON Schema files highly interoperable. While technically also a JSON file, a JSON Schema file adheres to a strictly defined hyperschema used to describe a metadata schema. The way I understand the manuscript, it is such JSON Schema files that are produced. If this understanding is correct, I would ask the authors to use the more precise term "JSON Schema". If this is understanding not correct, I would urge the authors to specify more precisely what is produced and add a comment if further steps are needed to make use of the output in the ELIXIR Biovalidator or other external validator tools.4 (only a comment, no change or response needed). I am very pleased that the authors have provided of "proof-of-concept" implementation of ontology term validation! This is, I believe, a very useful feature. My only feedback here is a suggestion that such validation could possibly be outsourced to the ELIXIR Biovalidator, which might simplify implementation. Of course, the devil is in the details, so that might not be a valid solution in practice.6 (minor review). The authors have now provided some comments on their experience and some concerns regarding the SEEK platform, after my mention of this. However those comments were only provided to the reviewers and not added to the manuscript

itself, as far as I can see. I see that I might not have been explicit about what I think is lacking in the manuscript: As a reader of the paper, I would very much like to see (preferably as part of the background) an overview of other published solutions to the problem the FAIR Data Station is aiming to solve, or some related tools if no directly overlapping tools exist. I would also like to see the authors compare their solution with the existing ones in more detail. The SEEK platform is one example of such a solution that I believe should be directly cited and discussed. The manuscript does mention a few other related tools, but only in passing and only in the conclusion, which I think is a bit late in the manuscript. Since many users are currently looking for FAIR data managements tools, such information would be useful even if one does not make use of the FAIR Data Station. This will also better place the paper in its scientific context.In conclusion, the authors have done a good job addressing the issues that I have raised. However, mainly due to issue #6, I think a minor review is still necessary. This is in any case a manuscript issue only, I do not see the need for additional implementations.

Level of Interest

Please indicate how interesting you found the manuscript: Choose an item.

Quality of Written English

Please indicate the quality of language in the manuscript: Choose an item.

Declaration of Competing Interests

Please complete a declaration of competing interests, considering the following questions:

- Have you in the past five years received reimbursements, fees, funding, or salary from an organisation that may in any way gain or lose financially from the publication of this manuscript, either now or in the future?
- Do you hold any stocks or shares in an organisation that may in any way gain or lose financially from the publication of this manuscript, either now or in the future?
- Do you hold or are you currently applying for any patents relating to the content of the manuscript?
- Have you received reimbursements, fees, funding, or salary from an organization that holds or has applied for patents relating to the content of the manuscript?
- Do you have any other financial competing interests?
- Do you have any non-financial competing interests in relation to this paper?

If you can answer no to all of the above, write 'I declare that I have no competing interests' below. If your reply is yes to any, please give details below.

I declare that I have no competing interests

I agree to the open peer review policy of the journal. I understand that my name will be included on my report to the authors and, if the manuscript is accepted for publication, my named report including any attachments I upload will be posted on the website along with the authors' responses. I agree for my report to be made available under an Open Access Creative Commons CC-BY license (http://creativecommons.org/licenses/by/4.0/). I understand that any comments which I do not wish to be included in my named report can be included as confidential comments to the editors, which will not be published.

Choose an item.

To further support our reviewers, we have joined with Publons, where you can gain additional credit to further highlight your hard work (see: https://publons.com/journal/530/gigascience). On publication of this paper, your review will be automatically added to Publons, you can then choose whether or not to claim your Publons credit. I understand this statement.

Yes Choose an item.
